# Complementary Medicine for Stimulation of Anticancer Immunity

**DOI:** 10.1155/2019/1316830

**Published:** 2019-05-26

**Authors:** Hyo-Jin An, Hongsheng Lin, Hwan-Suck Chung

**Affiliations:** ^1^Department of Pharmacology, College of Korean Medicine, Sangji University, Wonju, Republic of Korea; ^2^China Academy of Chinese Medical Sciences, Beijing, China; ^3^Korean Medicine (KM) Application Center, Korea Institute of Oriental Medicine (KIOM), Daegu, Republic of Korea

Although cancer cells are continuously generated in our body, immune surveillance system eradicates them so that we can stay healthy. However, sometimes the immune surveillance system cannot work properly; the cancer cells evade it and grow as a tumor burden [1]. Recently, cancer immunotherapy, which is not targeted for tumor cells but immune cells, has become an essential area for cancer treatment [2].

Various tumor-immunologic biologicals have been approved, and cancer immunotherapy has attracted great attention [3]. Even though there are many studies about immune regulation by complementary medicine, there are only a few studies of cancer immunotherapy by complementary medicine. Although complementary medicine has been used in clinic for cancer treatment, it is not clear yet from the stage of action mechanism. Even if it is experimentally effective, it is unknown whether it can really treat cancer by the mechanism. Therefore, there are many things to study in the future.

In this special issue, five studies were published regarding the complementary medicine for cancer immunotherapy. S. L. Oei et al. carried out a review about mistletoe,* Viscum album L.* (VA), which has a long traditional history of about 100 years as an add-on therapy of cancer treatment in German-speaking countries. They concluded that VA can exert apoptotic and cytotoxic as well as anti-inflammatory and immunological effects during cancer therapies. According to their review of previous studies, they supposed that VA may assist the elimination of cancerous cells and additionally supports standard oncological strategies by lowering adverse effects. T. Wang et al. compiled quantities of case history to evaluate the current available evidence of herbal Danshen (Radix Salviae Miltiorrhizae) formulae for the treatment of cancer by means of the high-quality randomized controlled trials (RCTs). Meta-analysis suggested that Danshen formulae had a significant effect on response rate, 1-year survival, 3-year survival, and 5-year survival. They concluded that that Danshen formulae combined with chemotherapy for cancer treatment was better than conventional drug treatment plan alone. X. You et el. analyzed the rule of kidney-tonifying method of Chinese medicine for the treatment of bone marrow suppression (BMS) in the Chinese medicine literature on databases including Chinese National Knowledge Infrastructure and Chinese Biomedical Literature Database. They showed that the treatment of BMS is mainly based on the method of invigorating the spleen, tonifying the kidney and liver to strengthen healthy qi, and supplementing with blood-activating herbs and dampness-draining diuretic herbs to eliminate pathogenic factors. Y. Chen et al. searched extracts and ingredients from five Qi-tonifying herbs exhibiting an inhibitory effect on M2 polarization of murine macrophages. Among them, total flavonoids from Glycyrrhizae Radix et Rhizoma suppressed M2 polarization of macrophages partly by inactivating STAT6 pathway and enhanced the level of miR-155 to regulate the expressions of M1 and M2 markers. D. Mao et al. evaluated the efficacy of Yanghe decoction (YHD) on antitumor and immune system enhancement in a 4T1 mouse breast cancer model. They showed that the mechanisms of YHD inhibiting 4T1 breast tumor growth may be related to downregulating the expression of iNOS and ARG-1, negatively regulating the Janus kinase/STAT3 (JAK/STAT3) pathway by repressing the expression of IL-6 and TGF-*β*. Meanwhile, YHD enhances the immune capacity via increasing the expression of NKTs, CD4+ T cells, IFN-*γ*, and p-STAT1. They provide new evidence of the effects of YHD on the treatment of breast cancer.

Most of the studies in this special issue focused on the complementary medicine in the cancer immunotherapy. These trials, clarifying the mechanism of effective complementary medicine in the cancer immunotherapy, can provide important and reasonable data to be applied to patients. We hope that readers will be interested in cancer immunotherapy using complementary medicine and that this special issue could be really special to the researchers studying and applying cancer immunotherapy.
